# Higher weight-adjusted waist index is associated with increased likelihood of kidney stones

**DOI:** 10.3389/fendo.2023.1234440

**Published:** 2023-09-20

**Authors:** Hangyu Liu, Yang Ma, Lungang Shi

**Affiliations:** ^1^ Department of Plastic Surgery and Burn Center, the Second Affiliated Hospital of Shantou University Medical College, Shantou, Guangdong, China; ^2^ Department of Plastic Surgery, Meizhou Clinical Institute of Shantou University Medical College, Meizhou, Guangdong, China

**Keywords:** weight-adjusted waist index, kidney stone, NHANES, obese, cross-sectional study

## Abstract

**Objectives:**

The objective of this study was to evaluate the association between weight-adjusted waist index (WWI) and the prevalence of kidney stones among adults in the United States.

**Methods:**

The cross-sectional study utilized data from the National Health and Nutrition Examination Survey (NHANES) spanning the years 2007-2018. A total of 31,344 participants were categorized into two groups: those with kidney stones and those without. WWI was determined by dividing waist circumference (cm) by the square root of body weight (kg). To examine the relationship between kidney stones and WWI, multivariate logistic regression models, smoothed curve fitting, and weighted generalized additive model (GAM) regression were employed. Subgroup analysis and interaction tests were conducted to explore the stability of this association across different groups.

**Results:**

The final analysis comprised a total of 31,344 participants, including 2,928 individuals who had a history of kidney stones. In the fully adjusted model, an increase in WWI exhibited a positive correlation with the prevalence of kidney stones (OR=1.34, 95% CI: 1.18-1.51). When WWI was converted into quartiles (Q1-Q4), participants in the highest quartile (Q4) had a 69% greater risk of developing kidney stones compared to those in the lowest quartile (Q1) (OR=1.69, 95% CI: 1.28-2.25). This positive association was particularly notable among non-diabetic patients.

**Conclusion:**

Our study demonstrates a significant positive association between weight-adjusted waist index levels and an elevated prevalence of kidney stones among US adults. Furthermore, this research highlights the potential utility of weight-adjusted waist index in the prevention of kidney stones in the overall population. This relationship is limited and further research is needed to test this hypothesis.

## 1 Introduction

Kidney stones (KS) is a disease caused by the formation of solid stones from solute deposits in the urinary tract, often presenting as painful renal colic or haematuria (1, 2). Research has indicated a significant rise in the prevalence and incidence of kidney stones in recent decades, with global estimates ranging from approximately 2% to 20%. It is estimated that kidney stones affect around 1–15% of the global population (2–5). Kidney stones may lead to irreversible damage such as ureteral blockage, urinary tract infection, cystitis, pyelonephritis or chronic pyelonephritis, and eventually end-stage renal failure (ESRF) (6). Most kidney stones require expensive surgical treatment (7), impose a significant medical burden on society as a whole and individuals, and have become a serious public health problem. Given the critical nature of the situation, enhancing early identification and preventive measures for kidney stones within the population becomes imperative.

Obesity represents a significant risk factor for kidney stone formation and substantially elevates the risk compared to individuals with normal weight. Existing studies indicate that the risk can be heightened by as much as 75% (8). In a comprehensive prospective cohort study examining the relationship between obesity, weight gain, and increased waist circumference, these factors were identified as novel risk factors for stone attacks in individuals with stone disease (9). Nevertheless, body mass index (BMI), waist circumference (WC), and waist-height ratio (WHtR), which are widely employed as indicators of obesity, possess inherent limitations, leading to increasing concerns and debate regarding their accuracy. The “obesity paradox” has emerged to varying degrees with inconsistent or contradictory results between these obesity indicators and mortality in different populations (10–12). Additionally, there exists a stronger correlation between body composition, body fat distribution, and unfavorable metabolic profiles. Conventional measures of obesity, such as body mass index (BMI) and waist circumference (WC), fail to differentiate between fat distribution and muscle mass (13).

As a result, researchers proposed a novel obesity index called the “weight-adjusted waist index(WWI) “ in their 2018 study (14). WWI is a novel biomarker that has recently been developed, with few pertinent studies, and its diagnostic and prognostic value is primarily in clinical studies in cardiovascular-related fields (15–17). WWI reduces the association with BMI and combines the benefits of waist circumference to better reflect body muscle and fat than traditional obesity metrics such as BMI. In addition, WWI shows greater stability compared to BMI and other factors, providing a more accurate representation of visceral fat and muscle mass, therefore, gaining interest (18, 19). Recent research indicates that WWI may also have implications for renal health. The findings indicate that the Weight-Adjusted Waist Index (WWI) can serve as a simple anthropometric index with the ability to accurately predict albuminuria (20). WWI, as a readily available and cost-effective measure of obesity, holds great potential for use across various healthcare settings, particularly in resource-limited areas or when extensive data analysis is required. In addition, WWI is linearly and positively correlated with cardiometabolic morbidity and mortality and has excellent predictive power in predicting cardiometabolic disease and cardiovascular disease (14), and in populations with high WWI, helping clinicians to assess target organ damage early and initiate treatment to minimize the risk of disease and improve prognosis. In a prospective cohort study, it was observed that weight-adjusted waist index (WWI) demonstrated superior predictive performance for cardiovascular disease (CVD) mortality when compared to BMI, WC, and waist-height ratio (WHtR). Notably, the association was found to be absent in the case of BMI and WC (14). The aforementioned studies propose the potential superiority of weight-adjusted waist index (WWI) as an obesity indicator. Nevertheless, the association between WWI and kidney stones remains uncertain.

Therefore, leveraging data acquired from the National Health and Nutrition Examination Survey (NHANES) spanning from 2007 to 2018, we explored the potential correlation between the WWI index and the probability of developing kidney stones. Our hypothesis posited that an elevated Weight-Adjusted Waist Index would be linked to an increased likelihood of kidney stones. To the best of our knowledge, this study is the first to investigate the correlation between WWI and the elevated prevalence of kidney stones.

## 2 Materials and methods

### 2.1 Study participants

**Figure 1:**
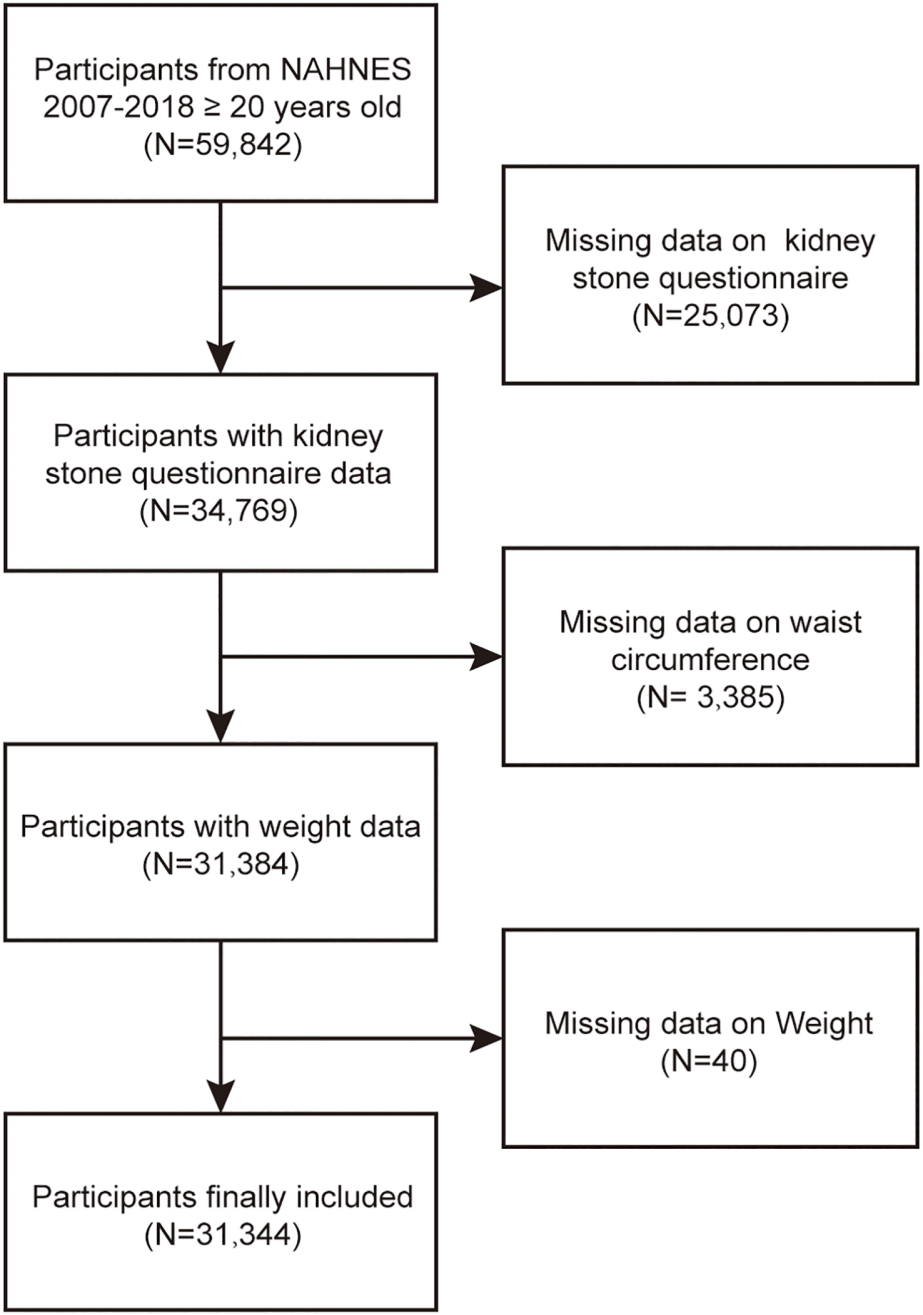
Flow chart of participants selection. NHANES, National Health and Nutrition Examination Survey.

The National Health and Nutrition Examination Survey (NHANES) is an extensive survey of the US population, employing intricate, multi-stage, and probabilistic sampling techniques to gather a comprehensive range of data on nutrition and health among the general population in the United States. This survey makes use of the NHANES dataset for the United States from 2007 to 2018. There were 59,842 participants in this round. After excluding individuals with missing laboratory and demographic information, the analysis encompassed a total of 31,344 subjects. **Figure 1** illustrates the flowchart depicting the process of sample selection. The overall protocol was approved by the ethical review committee at the National Center for Health Statistics (NCHS), and all participants provided informed consent by signing the consent form.

### 2.2 Exposure and outcome definitions

As an exposure variable, each participant’s WWI was calculated as the square root of waist circumference (cm) divided by body weight (kg). At baseline, this was recorded as a continuous variable. Kidney stone occurrence (variable name: KIQ 026) was designed as an outcome variable. The participant was considered to have a kidney stone if he or she answered yes to the question, “Have you or the sample person (SP) ever had a kidney stone?” Previous research has established the reliability of self-reported kidney stone status (21).

### 2.3 Study variables

The covariates chosen for this study were age, sex (male or female), race, education level, moderate activity, marital status, smoking status, diabetes, hypertension, alcohol consumption, household income to poverty ratio (household PIR), weight, Waist circumference, body mass index(BMI), total cholesterol, high-density lipoprotein cholesterol (HDL-C), and low-density lipoprotein cholesterol (LDL-C). These were based on prior knowledge of the weight-adjusted index and factors associated with kidney stones. The NHANES Survey Methodology and Analysis Guide offers in-depth details on the methods used to gather the variables. You can find more specific information on the aforementioned variables in the NHANES Methodology and Analysis Guide (https://wwwn.cdc.gov/nchs/nhanes/AnalyticGuidelines).

### 2.4 Statistical analysis

All statistical analyses in this study adhered to the guidelines set forth by the Centers for Disease Control and Prevention (CDC). Continuous variables were summarized using means and standard deviations, while categorical variables were presented as percentages. To evaluate differences between groups categorized by the presence or absence of kidney stones, weighted Student’s t-tests (for continuous variables) and weighted chi-square tests (for categorical variables) were employed. The independent association between the WWI index and kidney stones was investigated using multifactor logistic regression models. Three models were utilized: Model 1, without any adjustment variables; Model 2, adjusted for sex, age, and race; and Model 3, adjusted for all covariates except BMI, waist circumference, and weight, which exhibited a significant impact on the exposure factors. To further investigate the non-linear relationship between the WWI index and kidney stones, smoothed curve fitting and weighted generalized additive model (GAM) regression techniques were employed. To explore heterogeneous associations between study subgroups, stratified factors were analyzed in subgroups by sex, age, race, education level, smoking status, diabetes, and hypertension. The heterogeneity of associations between subgroups was assessed using a log-likelihood ratio test model, which included an interaction term. Statistical analyses were performed using R (version 4.2) and EmpowerStats (version 4.1), statistical computing, and graphing software. A p-value of less than 0.05 was deemed statistically significant.

## 3 Results

### 3.1 Characteristics of the study population

The study encompassed a total of 31,344 participants. **Table 1** presents the overall characteristics of the study population based on the presence or absence of kidney stones. Patients with kidney stones exhibited a mean age of 55.73 ± 16.15 years, with 55.70% being male. On the other hand, patients without kidney stones had a mean age of 48.72 ± 17.55 years, with 48.04% being male. Participants with kidney stones, when compared to the non-kidney stone population, exhibited a higher prevalence of male gender, non-Hispanic white ethnicity, sedentary lifestyle, marital or partnered status, higher frequency of smoking, lower alcohol consumption, lower HDL-C levels, elevated total cholesterol levels, increased waist circumference, and BMI levels, and higher prevalence of high blood pressure and diabetes. In this study, the mean WWI was 11.35 ± 0.79 for individuals with kidney stones and 11.05 ± 0.85 for those without kidney stones. WWI was significantly different between the two groups (p < 0.001).

**Table 1:** Baseline characteristics of participants between 2007 and 2018 (n=31344).

Characteristics	None-stone formers (N=28416)	Stone formers (N=2928)	*P*-value
Age (years)	48.72 ± 17.55	55.73 ± 16.15	<0.001
Sex, n (%)			<0.001
Male	13651 (48.04%)	1631 (55.70%)	
Female	14765 (51.96%)	1297 (44.30%)	
Race/ethnicity, n (%)			<0.001
Non-Hispanic White	11150 (39.24%)	1563 (53.38%)	
Non-Hispanic Black	6307 (22.20%)	391 (13.35%)	
Mexican American	4362 (15.35%)	378 (12.91%)	
Other race/multiracial	6597 (23.22%)	596 (20.36%)	
Education level, n (%)			0.609
Less than high school	6926 (24.37%)	738 (25.20%)	
High school	6507 (22.90%)	663 (22.64%)	
More than high school	14983 (52.73%)	1527 (52.15%)	
Moderate activities, n (%)			<0.001
Yes	11594 (40.80%)	1054 (36.00%)	
No	16822 (59.20%)	1874 (64.00%)	
Marital status ,n(%)			<0.001
Married or with partner	18859 (66.37%)	2141 (73.12%)	
Single	9557 (33.63%)	787 (26.88%)	
Smoked at least 100 cigarettes, n (%)			<0.001
Yes	12330 (43.39%)	1479 (50.51%)	
No	16086 (56.61%)	1449 (49.49%)	
Diabetes, n (%)			<0.001
Yes	3393 (11.94%)	639 (21.82%)	
No	24388 (85.82%)	2191 (74.83%)	
Borderline	635 (2.23%)	98 (3.35%)	
Hypertension, n (%)			<0.001
Yes	9734 (34.26%)	1461 (49.90%)	
No	18682 (65.74%)	1467 (50.10%)	
Average alcohol consumption (drinks/day)	4.22 ± 37.13	3.65 ± 33.81	<0.001
Family PIR	2.49 ± 1.63	2.48 ± 1.61	0.872
HDL-C (mg/dL)	53.21 ± 16.23	49.81 ± 14.63	<0.001
LDL-C (mg/dL)	113.66 ± 35.74	111.45 ± 34.21	0.201
Triglyceride (mg/dL)	124.11 ± 111.48	136.59 ± 105.77	<0.001
BMI (kg/m^2^)	29.01 ± 6.84	30.38 ± 6.81	<0.001
Waist circumference (cm)	98.91 ± 16.35	104.21 ± 16.24	<0.001
Weight-adjusted-waist index (cm/√kg)	11.05 ± 0.85	11.35 ± 0.79	<0.001

Mean ± SD for continuous variables: the P value was calculated by the weighted linear regression model. (%) for categorical variables: the P value was calculated by the weighted chi-square test.

Stone, Kidney stone; Family PIR, the ratio of family income to poverty; BMI, body mass index; HDL-C, High Density Lipoprotein cholesterol; LDL-C, Low-Density Lipoprotein Cholesterol.

### 3.2 Association between weight-adjusted waist index and kidney stones

**Figure 2:**
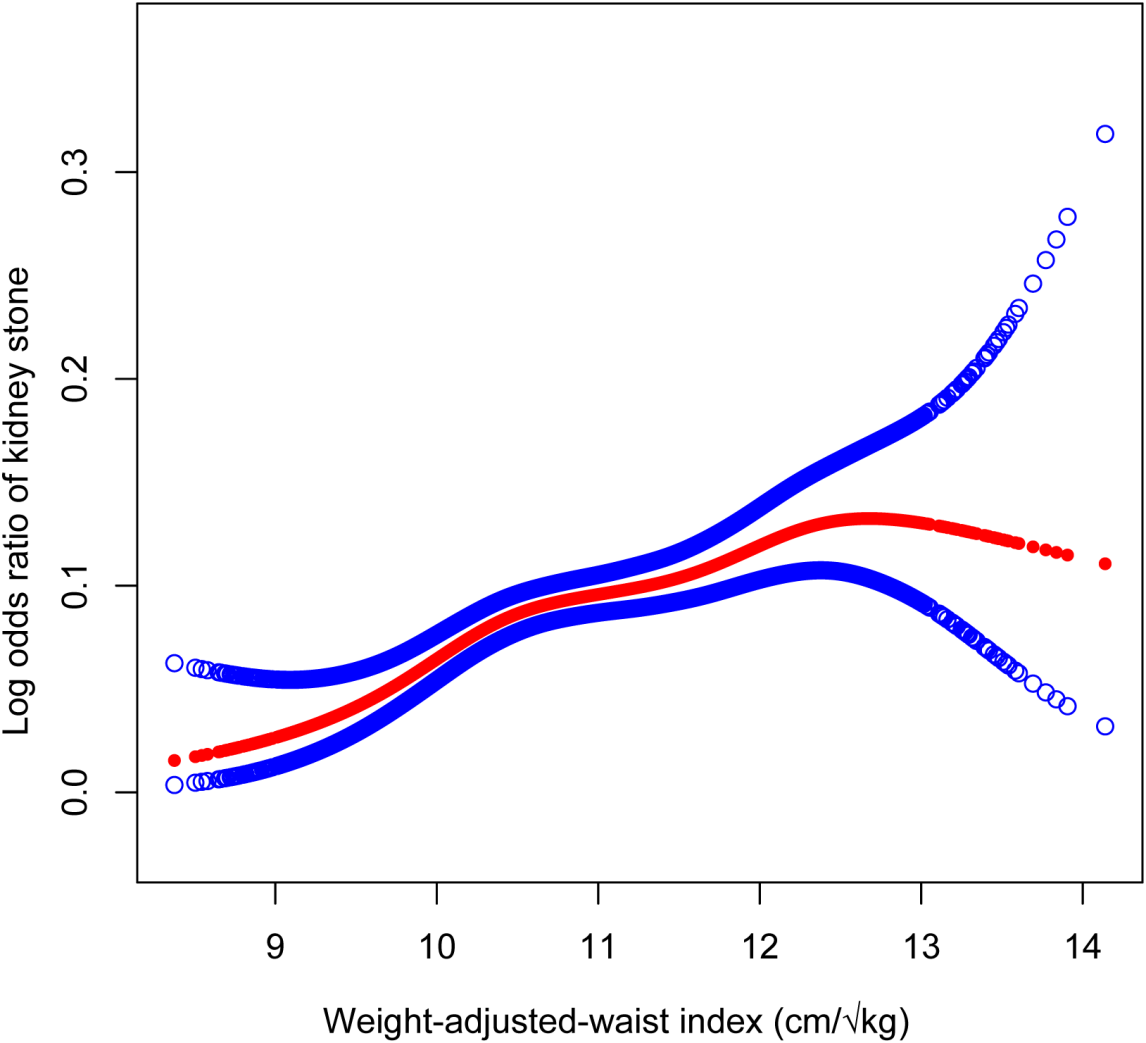
The association between Weight-Adjusted-Waist Index and Kidney Stones prevalence. Age, gender, race, educational level, marital status, HDL-C, LDL-C, triglyceride, PIR, moderate activities, diabetes status, hypertension status, smoking status and drinking alcohol status were adjusted.

**Figure 3:**
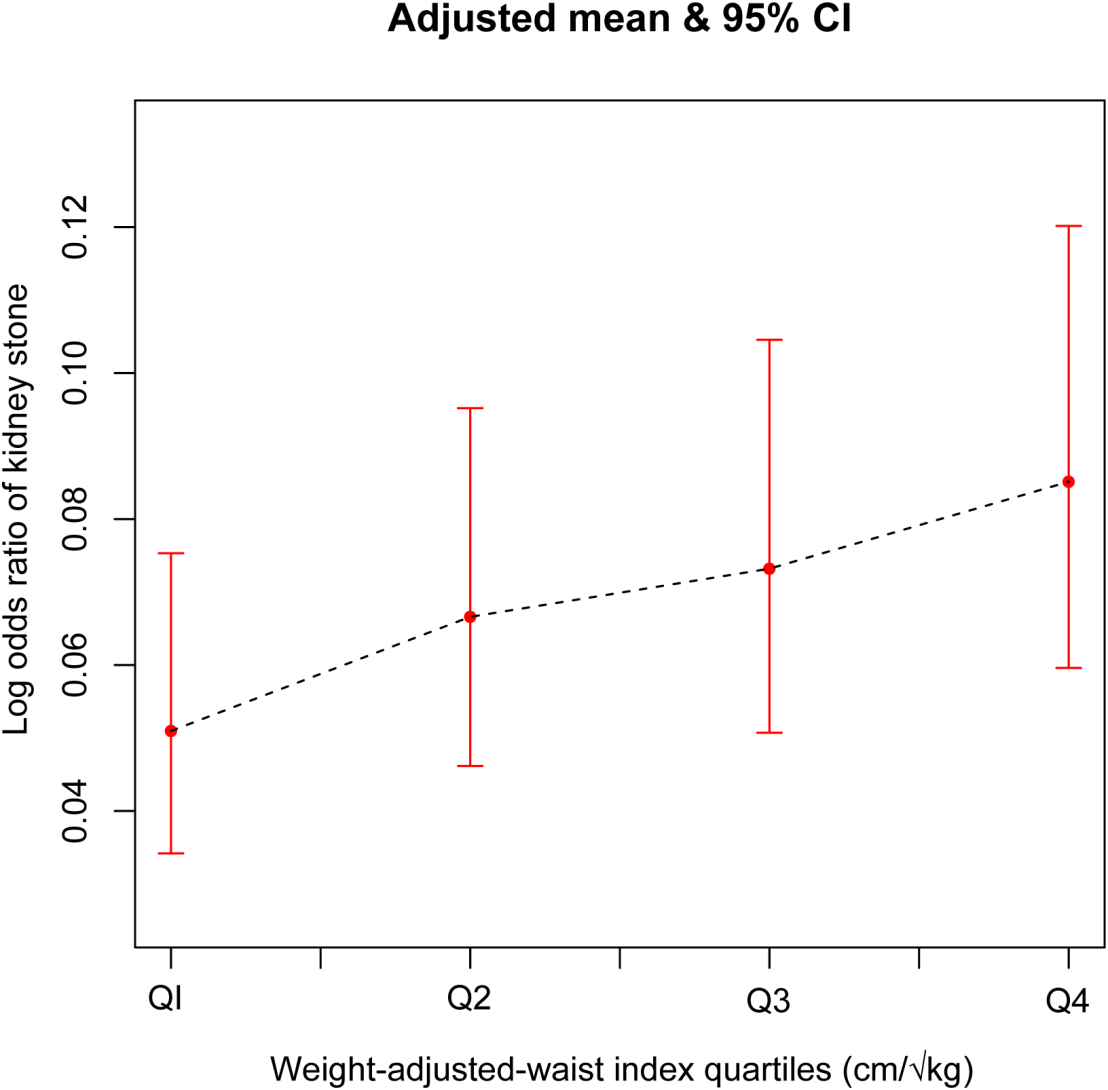
The association between Weight-Adjusted-Waist Index quartiles and Kidney Stones prevalence. Age, gender, race, educational level, marital status, HDL-C, LDL-C, triglyceride, PIR, moderate activities, diabetes status, hypertension status, smoking status and drinking alcohol status were adjusted.

In this study, as presented in **Table 2**, the unadjusted (Model 1) multifactorial logistic regression analysis revealed a positive association between WWI and the risk of kidney stones, with an odds ratio (OR) of 1.52 (95% CI: 1.45, 1.59). This association was consistently observed in Models 2 and 3 as well. In the fully adjusted model (Model 3) that accounted for all confounding factors, the correlation between WWI and kidney stones remained stable, with an odds ratio (OR) of 1.34 (95% CI: 1.18, 1.51). This suggests that for every unit increase in WWI, there was a 34% increased risk of developing kidney stones. Additionally, we obtained reliable findings even after categorizing the continuous variables into quartiles. The OR (95% CI) for Q2, Q3, and Q4 are displayed in **Table 2** and were 1.31 (1.02, 1.67), 1.44 (1.11, 1.88), and 1.69 (1.28, 2.25), respectively. The WWI index was 69% higher for Q4 than for Q1 (OR = 1.69, 95% CI: 1.28–2.25). indicating a potential non-linearity in this relationship. GAM and smoothing curves were employed to further explore the non-linear association between WWI and kidney stones, revealing a non-linear positive correlation (**Figures 2**, **3**).

**Table 2:** Association of weight-adjusted-waist index with kidney stone.

Exposure	OR (95% CI)		
	Model 1	Model 2	Model 3
	(n =31344)	(n=31344)	(n=8212)
WWI (m/√kg)	1.52 (1.45, 1.59)	1.39 (1.32, 1.47)	1.34 (1.18, 1.51)
WWI quartile			
Quartile 1	1.0	1.0	1.0
Quartile 2	1.59 (1.40, 1.80)	1.39 (1.22, 1.58)	1.31 (1.02, 1.67)
Quartile 3	2.11 (1.87, 2.38)	1.72 (1.51, 1.96)	1.44 (1.11, 1.88)
Quartile 4	2.62 (2.33, 2.95)	2.00 (1.75, 2.29)	1.69 (1.28, 2.25)
P for trend	<0.001	<0.001	<0.001

In sensitivity analysis, WWI was converted from a continuous variable to a categorical variable (quartile).

OR, odds ratio; 95% CI, 95% conﬁdence interval.

Model 1: No covariates were adjusted.

Model 2: Age, gender, and race were adjusted.

Model 3: Age, gender, race, educational level, marital status, HDL-C, LDL-C, triglyceride, PIR, moderate activities, diabetes status, hypertension status, smoking status and drinking alcohol status were adjusted.

PIR, the ratio of family income to poverty; BMI, body mass index; HDL-C, High Density Lipoprotein cholesterol; LDL-C, Low-Density Lipoprotein Cholesterol, WWI, weight-adjusted-waist index.

### 3.3 Subgroup analysis

**Figure 4:**
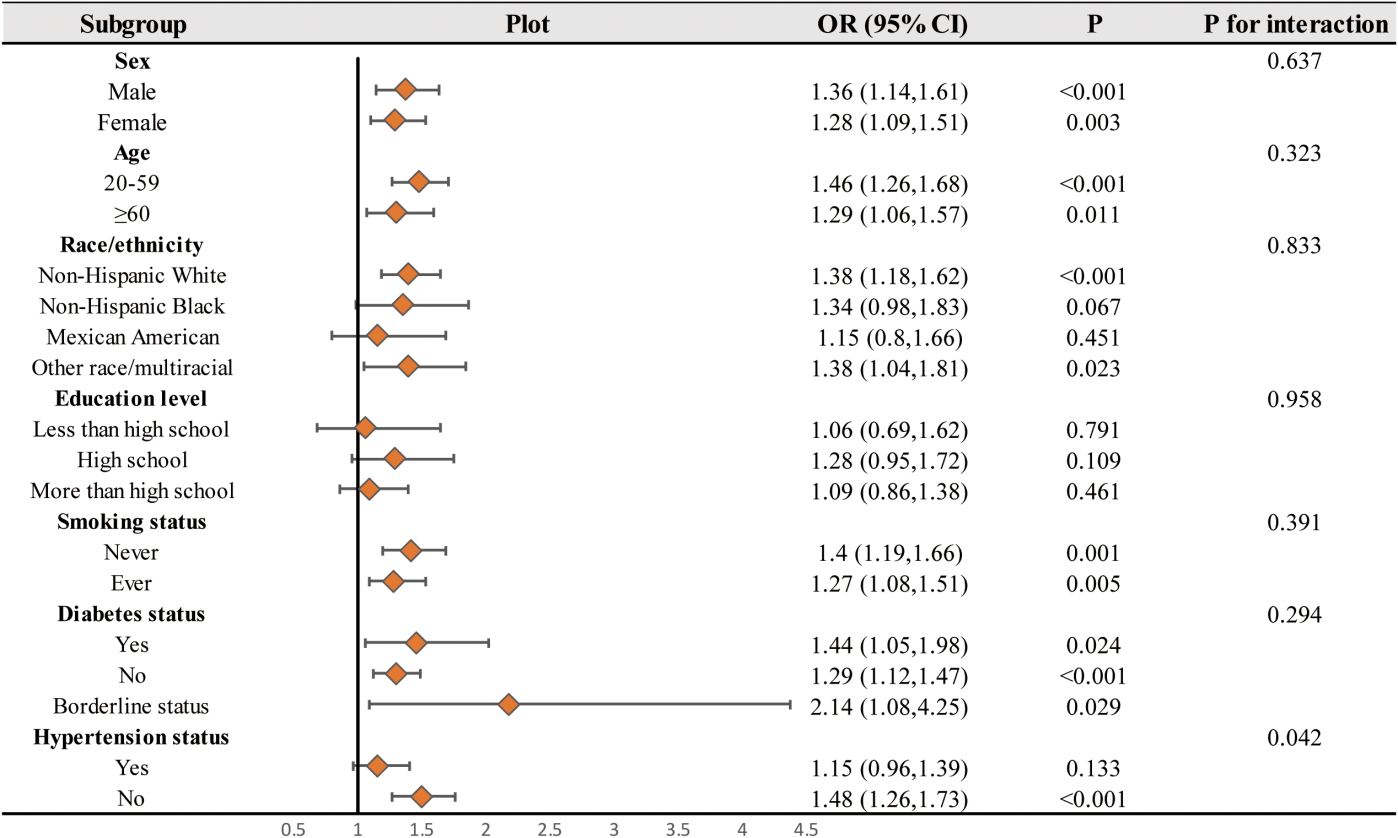
Subgroups analyses. In the subgroup analysis stratified by sex, age, Race/ethnicity, Education levels, Smoking status, Moderate activities, Diabetes status, and Hypertension status, the model is not adjusted for sex, age, Race/ethnicity, Education levels, Smoking status, Moderate activities, Diabetes status, and Hypertension status, respectively.

Subgroup analysis was conducted to assess the consistency of the correlation between WWI and kidney stones across different populations. Subgroup analysis (**Figure 4**) revealed that the correlation between WWI and the occurrence of kidney stones was not significantly influenced by age, gender, race/ethnicity, education level, smoking status, or diabetes status (p<0.05). Furthermore, we observed a significant interaction between the Hypertension status (Yes/No) groups and the correlation between WWI and kidney stones (interaction P < 0.05). In the non-hypertensive population, each 1-unit increase in WWI was associated with a 48% increase in the risk of kidney stones. However, in the hypertensive population, the correlation between WWI and the risk of kidney stones was found to be nonsignificant.

## 4 Discussion

The primary objective of this study was to evaluate the association between the weight-adjusted waist index (WWI) and the incidence of kidney stones. This cross-sectional study enrolled 31,344 adult participants over six consecutive NHANES 2-year cycles (2007–2018), and we observed an association between WWI and an increased likelihood of developing kidney stones. Subgroup analyses and interaction tests further demonstrated a significant dependence of hypertension (yes or no) and on this association. This suggests that higher levels of WWI may lead to an increased risk of developing kidney stones, particularly in those who are not hypertensive. The development and recurrence of kidney stones are known for their complexity. Understanding the risk factors associated with kidney stones is crucial for effective prevention and treatment strategies. Therefore, the weight-adjusted waist index (WWI) holds promise in the diagnosis of kidney stones.

Preliminary research has indicated the significant involvement of obesity in the development of kidney stones. Moreover, obesity has been shown to increase the risk of kidney stones, irrespective of whether the study participants have metabolic syndrome (22–24). A prospective study conducted by Taylor et al. investigated three large cohorts and found a significant association between obesity, weight gain, and the risk of kidney stone formation. Furthermore, the study revealed that women had a higher increased risk compared to men (25). In addition, Lee et al. discovered an association between obesity, metabolic changes, and the recurrence of urinary stones in their analysis of 704 patients, particularly in those who had previously formed stones (24). A retrospective study conducted by Kim et al. demonstrated a strong association between visceral fat obesity and uric acid stones. The study also indicated that visceral fat obesity had superior predictive value compared to BMI or urine pH in classifying stone type (26). A previous report indicated that the association between risk factors for uric acid stone formation and total body fat and trunk fat was stronger compared to body weight (27). The Android region, which includes the liver, pancreas, and kidneys, has been linked to an increased incidence of kidney stones, and an increase in the A/G ratio denotes an increase in the fat content of the Android region (21). On the other hand, Abufaraj et al. discovered that, even after controlling for various confounding variables, total body fat and trunk fat were associated with a significantly higher prevalence of kidney stones in adult women and men aged 40 years (28), and Bos et al. discovered that body fat distribution was also linked to the recurrence of kidney stones (29). Interestingly, earlier research has shown that WWI is a better predictor of body fat, especially concerning visceral fat (18). Similar to the findings of earlier studies, we discovered in this one that there is a correlation between the composite obesity index WWI and kidney stones, indicating that individuals with high levels of the weight-adjusted waist index (WWI) may be more likely to experience kidney stone formation. In addition, in our subgroup analysis, the association between WWI and kidney stones was more pronounced in non-hypertensive patients, and a series of earlier studies suggesting that hypertension (30, 31) is an independent risk factor for the development of kidney stones fits with previous findings.

One plausible explanation is that the accumulation of fat in the renal parenchyma may result in lipotoxicity. Specifically, the buildup of non-esterified fatty acids in renal cells, particularly in proximal tubular cells, could disrupt cellular metabolism, leading to a decreased production of ammonia. This reduction in ammonia output may contribute to renal damage, consequently promoting the development of kidney stones (32). On the other hand, it may be that increased fat is associated with elevated urinary oxalate, which contributes to the development of kidney stones through enhanced intestinal absorption of oxalate and increased endogenous synthesis of oxalate (26). In addition, obesity or fat gain can lead to inflammation and fibrosis of the kidney parenchyma or urinary tract infection, which is also a possible mechanism to induce the development of kidney stones (33–35).

This study has some advantages. It is a cross-sectional study using a broadly representative national representative sample. The study also employs a sophisticated multi-stage probability sampling design and makes extensive covariate adjustments, which significantly boosts the precision of statistical inference. In addition to the novel findings of the study, there are some shortcomings in our study. Firstly, as this study was cross-sectional, the relationship between WWI and kidney stones was limited. Also, more specific data were not included in the NHANES database, and due to the limitations of the questionnaire, such as the specific dose, specific type, site, and complications of kidney stones were information that we could not obtain. This affected the results to some extent. Thirdly, a lot of our data, including that on kidney stones, is self-reported and may be biased by recall. Given these limitations, further research is necessary. For example, study details such as the type of renal stone dose should be included to validate the findings of this study and to examine the relationship between WWI and renal stones in more detail.

## 5 Conclusion

Through the analysis of a nationally representative sample, this study revealed a significant association between high WWI and an increased incidence of kidney stones. We hypothesize that this has important implications for the prevention and treatment of kidney stones. Given the current sample of studies, this relationship is limited and this finding needs to be supported by further research to test this hypothesis.

## Data availability statement

The original contributions presented in the study are included in the article/supplementary material. Further inquiries can be directed to the corresponding author.

## Ethics statement

The studies involving humans were approved by National Center for Health Statistics (NCHS) Institutional Review Board. The studies were conducted in accordance with the local legislation and institutional requirements. The participants provided their written informed consent to participate in this study. The manuscript presents research on animals that do not require ethical approval for their study. Written informed consent was obtained from the individual(s) for the publication of any potentially identifiable images or data included in this article.

## Author contributions

HL and LS designed the research. HL, YM, and LS collected and analyzed the data. HL and YM drafted the manuscript. LS revised the manuscript. All authors contributed to the article and approved the submitted version.
